# Myocardial work index during normal dobutamine stress echocardiography

**DOI:** 10.1038/s41598-022-10903-8

**Published:** 2022-04-26

**Authors:** Marina Leitman, Yoni Balboul, Oleg Burgsdorf, Vladimir Tyomkin, Shmuel Fuchs

**Affiliations:** 1 Department of Cardiology, Shamir Medical Center, 70300 Zerifin, Israel; 2 Sackler School of Medicine, Tel Aviv University, Tel Aviv, Israel; 3 Department of Medicine, Icahn School of Medicine at Mount Sinai, Mount Sinai Morningside and Mount Sinai West, New York, NY USA

**Keywords:** Cardiovascular biology, Echocardiography, Cardiology

## Abstract

Dobutamine stress echocardiography is an alternative method to exercise stress echocardiography for the evaluation of ischemia. Recently, the novel speckle tracking imaging derived parameter, myocardial work index, was suggested for the evaluation of cardiac performance and was evaluated during exercise stress echocardiography. In this study, we analyzed the effect of dobutamine on myocardial work index variables during normal dobutamine stress echocardiography. Echocardiography examinations of patients with normal dobutamine stress echocardiography were collected and underwent off-line speckle tracking imaging analysis. Myocardial work index parameters were calculated at each dose of dobutamine and compared. 286 patients underwent dobutamine stress echocardiography during the study period. 102 patients were excluded due to pre-existed coronary artery disease or ischemia at dobutamine stress echocardiography. 65 patients were excluded due to suboptimal image quality unsuitable for speckle tracking imaging analysis. The remaining 119 patients with normal results were included. The global work index decreased from 2393.3 to 1864.7 mmHg%, p < 0.0004. Global constructive work decreased from 2681.7 to 2152.6 mmHg%, p = 0.001. Global wasted work increased from 78.8 to 128.3 mmHg%, p < 0.003. Global work efficacy decreased from 96.1 to 91.9%, p < 0.00001. Global strain increased from—19.6 to − 23.7%, p < 0.00001. Dobutamine stress echocardiography results in a decrease of all specific myocardial work index parameters even in normal subjects. Only global myocardial strain improved.

## Introduction

Dobutamine stress echocardiography (DSE) allows dynamic evaluation of cardiac function during pharmacological stimulation of heart rate, cardiac output, and myocardial oxygen demand. Diagnosis of ischemia is done by visual comparison of left ventricular contraction during dobutamine infusion. In contrast, to exercise stress echocardiography, dobutamine stress echocardiography isn’t expected to provide physiological information. Diagnostic accuracy of dobutamine stress echocardiography is determined by sensitivity for detection of coronary artery disease 61–96%, and specificity 70–100%^1^. Visual assessment of contractility is based on the evaluation of wall thickening that’s subjective and often completely depends on the experience of the interpreting physician. Recently, few studies evaluated cardiac mechanics during dobutamine stress echocardiography^2–4^ using speckle tracking imaging. The applicability of speckle tracking imaging in dobutamine stress echocardiography is limited by high heart rates. Recently a new speckle tracking imaging parameter, the myocardial work index was introduced^5^. To better understand the physiology of dobutamine stress echocardiography, we applied speckle tracking imaging, in particular a myocardial work index, in patients with normal dobutamine stress echocardiography.

## Materials and methods

Digital files of the patients that undergo dobutamine stress echocardiography examination were examined. All echocardiography exams were performed using Vivid E95, (General Electric) with a standard transducer of 1.7–4 Hz. Frame rate during dobutamine stress echocardiography examinations was in a range of 50–70 fps. Studies with normal results were selected. The clinical characteristics of these patients were obtained from the hospital files. Offline echocardiography analysis included a revision of conventional echocardiography and calculation of myocardial work index variables at baseline and each dose of dobutamine infusion. Speckle tracking imaging analysis was done according to the original recommendations from apical 3-chamber, 4-chamber, and 2-chamber views^6^. The myocardial work index variables included: Global work index (GWI), Global constructive work (GCW), Global wasted work (GWW), and Global work efficacy (GWE) and were calculated according to the recent recommendations^7^.

All the patients underwent a standard protocol of dobutamine stress echocardiography with the incremental rise of dobutamine doses every 3 min, from 10 up to 40 mcg/kg/min. Atropine was delivered at the 20 or 30 mg/kg/min dose of dobutamine as recommended^8^, particularly if the heart rate was not increased as expected. ECG and blood pressure measurements were registered every 3 min. The target heart rate was determined as 85% of maximal predicted heart rate, 85% × (220 − age).

Statistical methods. Continuous data were expressed as means ± standard deviations. The normal distribution for all differences was tested using the Kolmogorov–Smirnov test. A two-tailed, dependent T-test was used. Categorical data were expressed in numbers and percentages. Univariate analysis was performed using Chi-Square/Fisher’s exact test (where appropriate) or to identify significant variables (p < 0.05).

### Ethical approval

The study was approved by the Ethics (Helsinki) Committee at Shamir (Assaf Harofeh) Medical Center for the conduct of clinical Studies (No.: 0034-19-ASF). All methods were carried out following relevant guidelines and regulations.

Informed consent was not required as data collection was retrospective, anonymous, and speckle tracking imaging analysis didn’t influence clinical diagnosis. The Ethics (Helsinki) Committee at Shamir (Assaf Harofeh) Medical Center for the conduct of clinical Studies has approved the nonrequirement of informed consent**.**

## Results

During 01.2019–06.2019, dobutamine stress echocardiography examinations were performed in 286 patients. 102 patients were excluded due to a prior history of coronary artery disease or inducible ischemia at dobutamine stress echocardiography. 65 patients were excluded due to reduced echocardiography image quality, which wasn’t sufficient for adequate speckle tracking. The remaining 119 patients with normal dobutamine stress echocardiography were included in the current study. The mean age was 66, there were 38 males (32%). The main indications for the study were chest pain (53%) and shortness of breath (47%), while the most of patients had risk factors for coronary artery disease (Table 1).

**Table 1:** Demographic characteristics.

Number pts	119
Age	66.64 ± 11.32
Male/female	38/81 (32%/68%)
Beta blockers*	20 (16.8%)
BMI	30.57 ± 6.77
DM	35 (29%)
HPTN	98 (82%)
Hypercholesterolemia	99(83%)
PVD	32(27%)
Smoker	24(20%)
COPD	15(13%)
CVA	11(9%)
Chest pain	63(53%)
Dyspnea	56(47%)
EF	59.8 ± 1.57%
Atropine	53 (45%)
Atropine doses	0.7 ± 0.3
HR	89 ± 4%

*BMI* body mass index, *DM* diabetes mellitus, *HPTN* hypertension, *PVD* peripheral vascular disease, *COPD* chronic obstructive pulmonary disease, *CVA* cerebral vascular accident, *HR* heart rate.

*Beta blockers were stopped prior to DSE.

**Figure 1:**
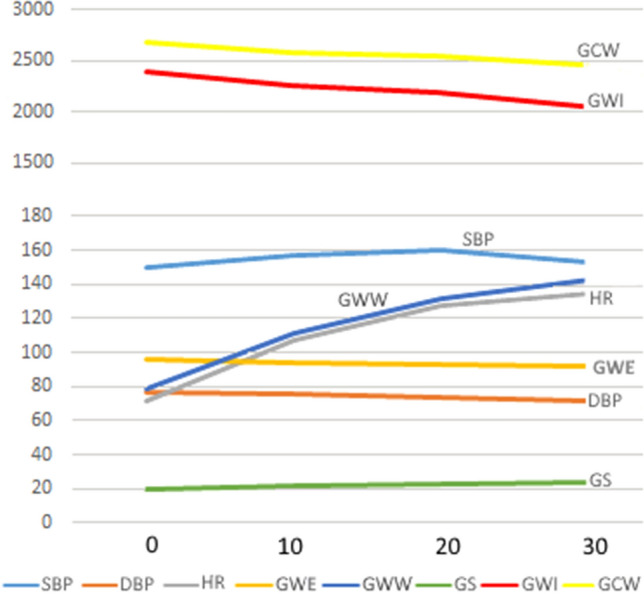
Changes in myocardial work index parameters during normal dobutamine stress echocardiography. *SBP* systolic blood pressure, *DBP* diastolic blood pressure, *HR* heart rate, *GS* global strain, *GCW* global constructive work, *GWW* global wasted work, *GWE* global work efficacy, *GWI* global work index.

The mean ejection fraction (EF) was 59.8%. 53 patients, 45%, received atropine at dobutamine dose 20 or 30 mcg/kg/min, mean atropine dose was 0.7 ± 0.3 mg. All the patients reached target heart rate: 13—at dobutamine doses of 40 mcg/kg/min, 79 at 30 mcg/kg/min, 27 patients reached target heart rate at dobutamine dose 20 mcg/kg/min. Peak heart rate was 89 ± 4% of maximal predicted heart rate. Myocardial work index parameters during each stage of dobutamine stress echocardiography and hemodynamic parameters are represented (Table 2, Fig. 1).

**Table 2:** Myocardial work index parameters during normal dobutamine stress echocardiography.

	Baseline	10 mcg/kg/min	20 mcg/kg/min	30 mcg/kg/min	40 mcg/kg/min
SBP, mmHg	149.7 ± 22.7	156.9 ± 26.6 *p* = 0.02	159.9 ± 28.9 *p* < 0.003	153.3 ± 26.8 *p* = 0.32	140.6 ± 14.6 *p* = 0.15
DBP, mmHg	76.3 ± 12.2	75.6 ± 14.1 *p* = 0.7	73.2 ± 15.9 *p* = 0.09	71.9 ± 15.8 *p* = 0.006	65.6 ± 16.6 *p* = 0.007
HR, bpm	71.9 ± 12.2	107.2 ± 18.4 *p* < 0.00001	127.1 ± 13.8 *p* < 0.00001	134.8 ± 11.6 *p* < 0.00001	133.4 ± 12.1 *p* < 0.00001
GS, %	− 19.6 ± 2.4	− 22.1 ± 2.8 *p* < 0.00001	− 23.0 ± 2.8 *p* < 0.00001	− 23.8 ± 2.8 *p* < 0.00001	− 23.7 ± 3.5 *p* < 0.00001
GWI, mmHg%	2393.3 ± 503.4	2262.0 ± 572.8 *p* = 0.06	2189.8 ± 568.1 *p* < 0.004	2060.6 ± 565.2 *p* = 0.00001	1864.7 ± 424.4 *p* < 0.0004
GCW, mmHg%	2681.7 ± 549. 6	2581.6 ± 660.1 *p* = 0.2	2551.9 ± 691.6 *p* = 0.74	2469.9 ± 717.6 *p* = 0.02	2152.6 ± 521.7 *p* = 0.001
GWW, mmHg%	78.8 ± 56.5	110.9 ± 53.1 p < 0.00001	131.0 ± 65.6 *p* < 0.00001	142.4 ± 73.7 *p* < 0.00001	128.3 ± 45.2 p < 0.003
GWE, %	96.1 ± 3.1	94.2 ± 2.8 *p* < 0.00001	93.0 ± 3.4 *p* < 0.00001	92.3 ± 3.7 *p* < 0.00001	91.9 ± 4.5 *p* < 0.00001

**Figure 2:**
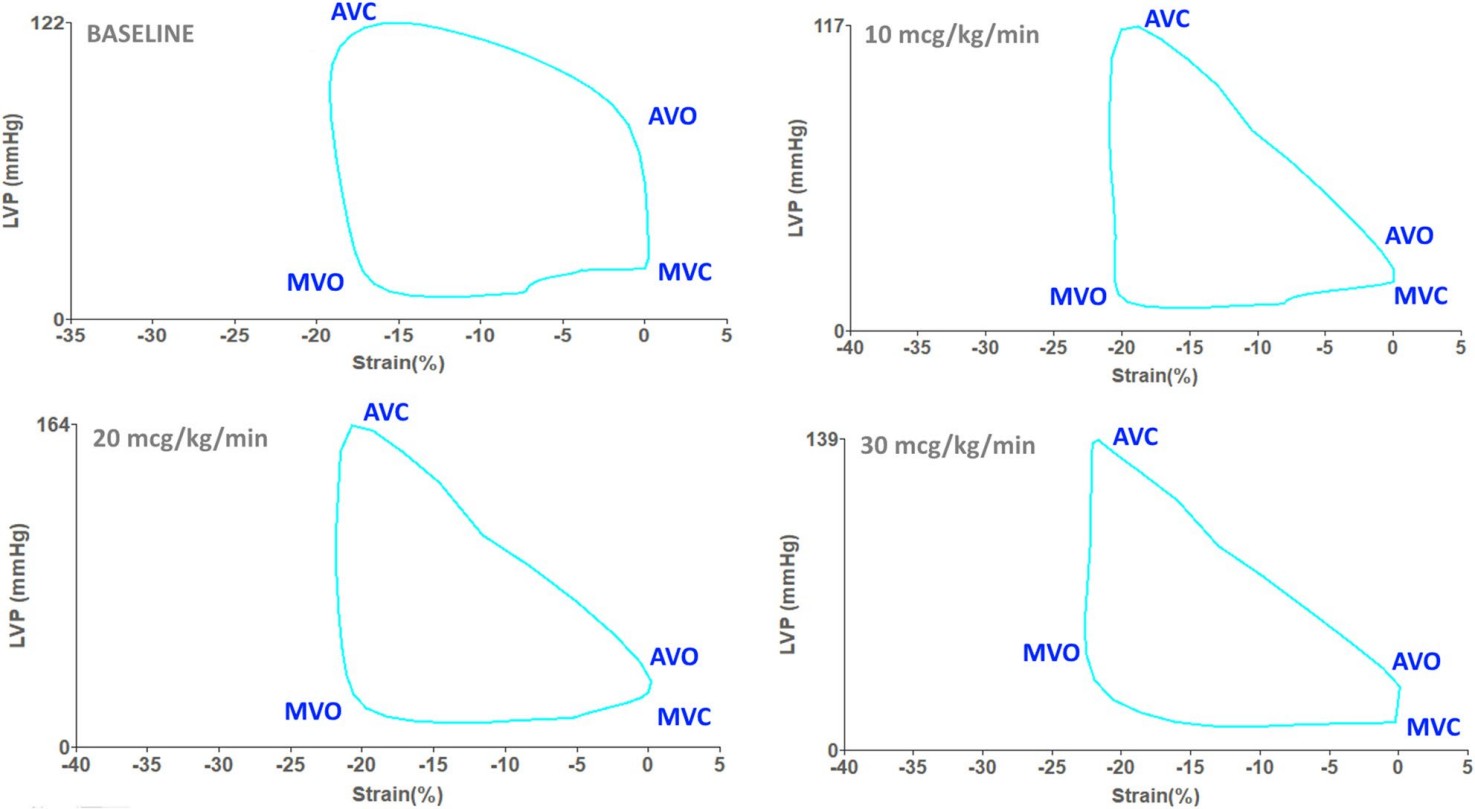
Pressure-strain loops during normal dobutamine stress echocardiography. *LVP* left ventricular pressure. *MVC* mitral valve closure, *MVO* mitral valve opening, *AVC* Aortic valve closure, *AVO* aortic valve opening. A small rise of strain results in a steeper increment of LV pressure during dobutamine stress echocardiography.

Systolic blood pressure reached a peak at dobutamine doses of 20 mcg/kg/min. Diastolic blood pressure decreased slightly from 76.3 ± 12.2 to 65.6 ± 16.6 mmHg, p = 0.007. Heart rate increased from 72 to 135 bpm, p < 0.00001. Global strain increased from − 19.6 to − 23.7%, p < 0.00001. Global work index (GWI) decreased from 2393.3 ± 503.4 to 1864.7 ± 424.4 mmHg%, p < 0.0004. Global constructive work (GCW) decreased from 2681.7 ± 549.6 to 2152.6 ± 521.7 mmHg%, p = 0.001. Global wasted work (GWW) increased from 78.8 ± 56.5 to 128.3 ± 45.2 mmHg%, p < 0.003. Global work efficacy (GWE) decreased from 96.1 ± 3.1 to 91.9 ± 4.5%, p < 0.00001. Changes in the strain pressure loops during dobutamine stress echocardiography show (Fig. 2) that a small increase in strain during the cardiac cycle results in a steeper elevation of left ventricular pressure during the increment of dobutamine doses.

The data were analyzed by ML. Intraobserver variability [ML and VT], was performed on 10 patients and ranged up to 5%. Interobserver variability [ML] was done on 10 patients and ranged up to 5%.

## Discussion

Dobutamine stress echocardiography is considered a good pharmacological alternative to stress echocardiography, within a generally acceptable safety profile^9^. Despite it, 7.6% of studies are stopped prematurely because of side effects^10^. Accelerated protocols of short-term high dose dobutamine infusion were suggested^11^ in the past. Modern protocols with atropine delivery at 20 and 30 mcg/kg/min were developed more recently to shorten the study and to improve its tolerability^8^. The various effects of dobutamine during dobutamine stress echocardiography are dose-dependent. Dobutamine has an affinity for cardiac α- and β-receptors. β1-receptors stimulation results in an increase in myocardial contractility, atrioventricular conduction, and heart rate. Stimulation of α-adrenergic receptors results in systemic vasoconstriction, increased myocardial contractility, and raises blood pressure. Stimulation of vascular β_2_-receptors induces coronary and peripheral arteriolar vasodilatation. The inotropic effect of dobutamine predominates in low doses, and the chronotropic effect develops in high doses^12^. Low dose dobutamine of 2.5–10 mcg/kg/min, is used for the assessment of viability^8^. Ischemic protocols of dobutamine stress echocardiography begin with the doses of 10 mcg/kg/min. The majority of patients have experienced a rise in systolic blood pressure, especially patients with preexisting hypertension that are more susceptible to the hypertensive effect of dobutamine^13^. 82% of our patients were hypertensive and dobutamine infusion resulted in a rise in blood pressure (Fig. 1), which is one of the major determinants of myocardial work index parameters.

Mechanics of cardiac contraction was evaluated recently with novel speckle tracking derived parameter, myocardial work index^5,14–17^. Evaluation of cardiac deformation parameters at stress echocardiography imaging up to recently was limited due to suboptimal speckle tracking imaging quality and to the inability to track endocardial border at high heart rates. Visual and quantitative analysis of left ventricular function, calculation of strain, and myocardial work index during high heart rates more than 120/min is problematic on the echocardiography monitors with fixed [non-adaptive] refresh rate, as some frames are distorted or explained twice or dropped. Recently introduced monitors with a high adaptive refresh rate allow an adequate solution to this problem^18,19^. As well, a better quality of modern echocardiography systems makes echocardiography images more suitable for speckle tracking imaging analysis.

Few studies evaluated strain during exercise stress echocardiography^20^ and dobutamine stress echocardiography^5,17–23^. A novel myocardial work index was investigated in patients during exercise stress echocardiography^24–26^, and in athletes after a half marathon^27^. During normal exercise stress echocardiography, deterioration was found only in global wasted work^25^ and global work efficacy^25,27^; the global work index improved^25,27^ and global constructive work improved^25^. In one study^26^, myocardial work index parameters were compared in normal versus ischemic response. In normal subjects, global longitudinal strain, global work index, global constructive work improved, global wasted work increased, global work efficacy was preserved [96% at rest, 95% at exercise]. In our study, despite improvement in global strain, all the parameters of myocardial work deteriorated during dobutamine stress echocardiography: global work index, global constructive work, global work efficacy, and global wasted work (Table 3). Therefore, the cardiac influence of dobutamine on myocardial work index variables is different from the effect of exercise. Dobutamine stress echo and exercise stress echo, both stress tests, affect conventional echocardiography parameters differently.

**Table 3:** Cardiac effects of dobutamine and exercise stress echocardiography.

Cardiovascular parameters	DSE*	ESE**
Heart rate	↑	↑
Systolic blood pressure	↑	↑
Global strain	↑	↑
Global work index	↓	↑
Global constructive work	↓	↑
Global wasted work	↑	↑
Global work efficacy	↓↓	↓

*DSE* dobutamine stress echocardiography, *ESE* exercise stress echocardiography.

*Data regarding DSE—current study.

**Data regarding ESE—literature data Halabi et al.^23^, Borrie et al.^24^.

In addition to increases in heart rate, systolic blood pressure, and global strain, other indicators of myocardial function are impaired during dobutamine stress echocardiography. End diastolic volume index decreases slightly with exercise and decreases significantly with dobutamine, end-systolic volume index decreases with exercise and decreases significantly with dobutamine, stroke volume decreases significantly during dobutamine infusion and increases with exercise^28^. Myocardial work index parameters during dobutamine stress echocardiography also behave differently than exercise stress echocardiography (Table 3).

Calculation of target heart rate during exercise stress test based on the real data about oxygen consumption response to exercise, that is linear. A stage of the plateau is reached at near maximal exercise when a heart rate is of about 220 − age, that is the maximal predicted heart rate^29^, therefore target heart rate during the exercise stress test is set as 85–90% × (220 − age) according to the current guidelines^8^. Oxygen consumption during dobutamine stress echocardiography is not known, but extrapolating the data from exercise echocardiography and for safety considerations, the target heart rate is empirically set as 85% of maximal predicted heart rate^8^.

Our results indicate that the dobutamine stress test can overload the heart and age-related heart rate may not be the optimal criterion for interrupting the dobutamine infusion that probably, can be terminated earlier. There is a question if the sensitivity of such a study will not be impaired and further evaluation is needed.

### Limitations

1. Only normal examinations were recruited into the study, therefore the researchers were not blinded to the results.
2. In our study we compared our data of dobutamine stress echocardiography with the literature data regarding exercise stress echocardiography that was done on other patients’ populations.
3. Although all our patients achieved a target heart rate, we can’t exclude the possibility of false-negative results.

## Conclusions

As we found in our study, during normal dobutamine stress echocardiography, although global myocardial strain increases, all the specific parameters of myocardial work index deteriorate. We can suppose, that the calculated target heart rate may not be the optimal parameter to define termination of dobutamine infusion. Myocardial work index and global strain can be used too. Further investigations of myocardial work index variables in patients with ischemia are needed.
